# Vesivirus 2117 capsids more closely resemble sapovirus and lagovirus particles than other known vesivirus structures

**DOI:** 10.1099/jgv.0.000658

**Published:** 2017-03-17

**Authors:** Michaela Conley, Edward Emmott, Richard Orton, David Taylor, Daniel G Carneiro, Kazuyoshi Murata, Ian G Goodfellow, Grant S Hansman, David Bhella

**Affiliations:** ^1^​ Medical Research Council – University of Glasgow Centre for Virus Research, Sir Michael Stoker Building, Garscube Campus, 464 Bearsden Road, Glasgow G61 1QH, UK; ^2^​ Department of Pathology, Division of Virology, University of Cambridge, Addenbrooke's Hospital, Hills Road, Cambridge CB2 2QQ, UK; ^3^​ National Institute for Physiological Sciences (NIPS), 38 Nishigonaka, Myodaiji, Okazaki, Aichi 444-8585, Japan; ^†^​ Present address: Howard Hughes Medical Institute, 742 Stanley Hall, MS 3220 University of California, Berkeley, CA 94720-3220, USA.; ^‡^​ Present address: School of Immunity and Infection, Institute of Biomedical Research, University of Birmingham, Edgbaston, Birmingham B15 2TT, UK.; ^§^​ Present address: Centre for Infectious Diseases, Department of Virology, University Hospital Heidelberg, Im Neuenheimer Feld 324, Heidelberg 69120, Germany.

**Keywords:** calicivirus, capsid, virus structure, cryo-EM

## Abstract

Vesivirus 2117 is an adventitious agent that, in 2009, was identified as a contaminant of Chinese hamster ovary cells propagated in bioreactors at a pharmaceutical manufacturing plant belonging to Genzyme. The consequent interruption in supply of Fabrazyme and Cerezyme (drugs used to treat Fabry and Gaucher diseases, respectively) caused significant economic losses. Vesivirus 2117 is a member of the *Caliciviridae*, a family of small icosahedral viruses encoding a positive-sense RNA genome. We have used cryo-electron microscopy and three-dimensional image reconstruction to calculate a structure of vesivirus 2117 virus-like particles as well as feline calicivirus and a chimeric sapovirus. We present a structural comparison of several members of the *Caliciviridae*, showing that the distal P domain of vesivirus 2117 is morphologically distinct from that seen in other known vesivirus structures. Furthermore, at intermediate resolutions, we found a high level of structural similarity between vesivirus 2117 and *Caliciviridae* from other genera: sapovirus and rabbit hemorrhagic disease virus. Phylogenetic analysis confirms vesivirus 2117 as a vesivirus closely related to canine vesiviruses. We postulate that morphological differences in virion structure seen between vesivirus clades may reflect differences in receptor usage.

## Abbreviations

3D, three-dimensional; CHO, Chinese hamster ovary; cryo-EM, cryogenic electron microscopy; EM, electron microscopy; FCV, feline calicivirus; NoV, Norwalk virus; RHDV, rabbit hemorrhagic disease virus; SMSV, San Miguel sea lion virus; SV, sapporovirus; VESV, vesicular exanthema of swine virus; VLP, virus-like particle.

## Introduction

Caliciviruses are non-enveloped icosahedral viruses that have single-stranded, positive-sense RNA genomes. The *Caliciviridae* are divided into five genera, namely, *Norovirus*, *Sapovirus*, *Vesivirus*, *Lagovirus* and *Nebovirus.* The representative viruses of these genera are Norwalk virus (NoV), sapporovirus (SV), feline calicivirus (FCV), rabbit hemorrhagic disease virus (RHDV) and Newbury-1 virus, respectively. The noroviruses and sapoviruses cause gastroenteritis in humans, while neboviruses have been shown to cause gastroenteritis in cattle. Vesiviruses and lagoviruses cause a range of symptoms in different animal species including stomatitis, conjunctivitis, respiratory illness and hemorrhagic disease [1–3].

Calicivirus genomes are around 7.5 kb in length, encode up to four ORFs and are polyadenylated. The first ORF encodes the non-structural proteins and in the lagoviruses and sapoviruses the major capsid protein, VP1. In the genera *Norovirus* and *Vesivirus*, the VP1 protein is encoded by a second ORF and the minor structural protein, VP2, is encoded by ORF3, which is translated following ribosomal termination–reinitiation [4]. The ORF1 polyprotein is post-translationally cleaved by the autocatalytic viral protease to yield several non-structural proteins [5]. The major capsid protein, VP1, is also post-translationally processed, resulting in the removal of the N-terminal 124 amino acids to produce the mature form of the protein (62 kDa) [6]. Murine norovirus (MuNV) is the only member of the *Caliciviridae* that encodes a fourth ORF, the product of which, referred to as VF1, is involved in the regulation of the innate response to infection [7].

Members of the *Caliciviridae* exhibit a characteristic virion morphology of 32 cup-shaped depressions on their outer surface. The *T*=3 capsid is composed of three quasi-equivalent forms of the VP1 protein, termed A, B and C. These give rise to two classes of dimeric capsomere: A/B and C/C. Ninety dimers/capsomeres assemble to form a 35–40 nm spherical capsid that encloses the viral genome. A/B and C/C capsomeres differ only slightly in their conformations. An atomic model of RHDV shows the A/B capsomeres (described as bent) arranged around the fivefold symmetry axis, while the C/C capsomere, which adopts a flatter conformation, is located at the twofold symmetry axis [8–11]. The VP1 protein has been divided into three domains: the N-terminal arm, the shell domain (S) and the protruding domain (P). The N-terminal arm is hypothesized to be involved in the switch between the A/B and C/C conformations. The S domain contains a β-barrel motif and forms the floor of the viral capsid. Sequences within the S domain have been shown to interact with the viral RNA-dependent RNA polymerase to promote *de novo* RNA synthesis [12]. The P domain is further divided into two subdomains, P1 and P2, with the P2 domain containing the antigenic and receptor-binding sites [13–15].

Recently, vesivirus 2117 was identified as a contaminant of bioreactors containing Chinese hamster ovary (CHO) cells at Genzyme in both their Allston Landing and Geel sites. This led to an interruption in production of Cerezyme and Fabrazyme biopharmaceuticals for the treatment of patients suffering from Gaucher disease and Fabry disease, respectively. Vesivirus 2117 was first described as an adventitious agent from an unknown source following observations of cytopathic changes in CHO cells grown in culture [16]. Electron microscopy (EM) showed the presence of viral particles measuring approximately 40 nm in diameter and exhibiting the typical morphology of caliciviruses. Upon infection of CHO cells with vesivirus 2117, cells become rounded and detach from cell culture plastic within 24 h, which accounts for the loss of cell viability seen at Genzyme. It is thought that the viral contamination at Genzyme was introduced from reagents used in the manufacturing process. It was estimated that approximately 5×10^9^ virus particles were present in each millilitre of bioreactor fluid. Subsequently, the bioreactors were closed for decontamination, causing a significant delay in the production of Cerezyme and Fabrazyme, which affected around 8000 patients. Stock analysts estimate that the contamination and interruption in manufacturing may have cost Genzyme in the region of $200–300 million [17].

Adventitious agent testing is mandated for the prevention of contamination by viruses able to replicate in CHO cells and has led to the detection of five incidents in the past 20 years caused by murine minute virus, reovirus, Cache Valley virus, epizootic hemorrhagic disease virus and vesivirus 2117 [18–23]. Currently, three processes are adopted to minimize the risk of introducing contaminants into cell culture: control and testing of raw materials, testing at key stages during manufacturing and the use of virus inactivation techniques. These steps are necessary owing to the extent to which CHO cells in particular are used for the production of therapeutic agents. Although these cells are less permissive to infection than, for example, baby hamster kidney cells, episodes of contamination have been documented. In each case, the source of the contaminants was suspected to be input raw materials. Since 1998, the Food and Drug Administration has made it a regulatory requirement in the production of biopharmaceuticals to demonstrate the lack of adventitious agents [18, 20, 24, 25].

Another calicivirus, SV (the type member of the genus *Sapovirus*), was first identified by EM in 1976 from stool samples of infants presenting with gastroenteritis. Sapovirus strains are classified into five genogroups (GI–GV) based on their capsid protein sequences. Currently, only GIII viruses can be propagated in cell culture; however, members of this genogroup cause porcine infections, while the other genogroups cause gastroenteritis in humans. As is the case for many members of the *Caliciviridae*, expression of a recombinant form of the major capsid protein is sufficient for the assembly of virus-like particles (VLPs), which exhibit the same morphology as virions [26–29].

Here we describe the structures of VLPs formed by recombinant expression of vesivirus 2117 VP1 and a chimeric sapovirus VP1, alongside an improved structure for FCV strain F9, all solved by cryogenic EM (cryo-EM) and three-dimensional (3D) image reconstruction. These data reveal that the vesivirus 2117 capsid structure more closely resembles that of sapoviruses, and surprisingly a lagovirus, than it does other known vesivirus structures. Phylogenetic analysis of capsid protein sequences supports the classification of vesivirus 2117 as a member of the genus *Vesivirus*, although it resides in a clade distinct from FCV and the vesicular exanthema of swine virus (VESV) and San Miguel sea lion virus (SMSV) groups. It has recently been proposed that structural similarities may be used to infer common heritage of diverse virus groups in the absence of genetic similarity [30]. Here we see substantial differences in virion morphology between clades of the genus *Vesivirus* and, albeit at comparatively low resolution, similarities between vesivirus 2117 and structures for both sapoviruses and lagoviruses. The striking differences in P domain morphology within vesiviruses may indicate functional differences, perhaps related to receptor usage and entry mechanisms.

## Results

### Vesivirus 2117 VLPs are structurally distinct from known vesivirus capsid structures

**Fig. 1.**
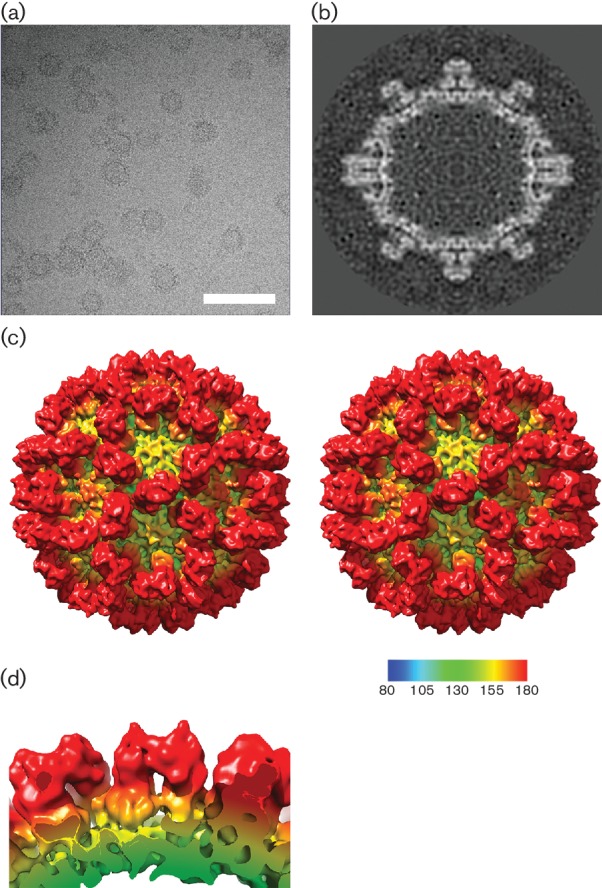
Cryo-EM structure of vesivirus 2117 at 10 Å resolution. (a) Cryo-electron micrograph of vesivirus 2117 VLPs imaged in a frozen-hydrated state. Bar, 100 nm. (b) A central slice through the 3D reconstruction of vesivirus 2117 shows the compact structure of the P domain. (c) Stereo pair images of the reconstruction, calculated at 10 Å resolution, viewed along the twofold symmetry axis. (d) A side view of the 2117 VP1 dimer viewed parallel to the capsid surface highlights the pronounced horn-shaped structures on the outer faces of the P domains.

Vesivirus 2117 VLPs were prepared by baculovirus expression of VP1 in Hi5 insect cells and purified by differential centrifugation. VLP preparations were vitrified by plunge freezing in liquid ethane and imaged in a frozen-hydrated state by cryogenic transmission electron microscopy (Fig. 1a). Nine hundred and sixty particle images were extracted from 242 micrographs and processed to calculate a 3D icosahedral reconstruction of the VLP at a resolution of 10 Å (Fig. 1b, c). Vesivirus 2117 VLPs were seen to exhibit the characteristic calicivirus morphology: a *T*=3 icosahedral capsid composed of VP1 dimers, giving rise to arch-like capsomeres. The VP1 dimers also appear to form intradimeric interactions between P2 domains and interdimeric interactions between P1 domains, similar to those seen in FCV and as previously described for SMSV [31]. Viewed perpendicular to the capsid floor, a side view of the VP1 dimeric capsomere (Fig. 1d) shows that the outer surface of the P2 domain has pronounced horn-like structures at either end. This is quite distinct from the morphology seen in previously solved vesivirus capsid structures [13, 14, 32]. This morphological divergence from known vesivirus capsid structures led us to hypothesize that 2117 might represent an intermediate between classical vesiviruses and other genera. We therefore set out to compare 2117 VLPs to other calicivirus capsid structures.

### Comparison of vesivirus 2117 VLP structure to that of a chimeric sapovirus

**Fig. 2.**
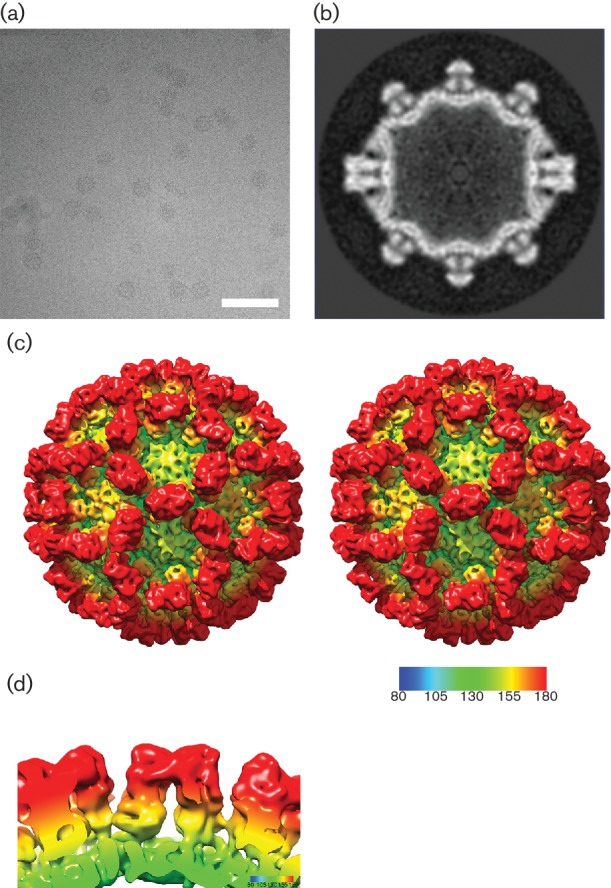
3D reconstruction of a chimeric sapovirus VLP. (a) Cryo-electron micrograph of chimeric sapovirus-like particles. Bar, 100 nm. (b) A central slice through the sapovirus VLP structure reveals a compact P domain structure, similar to that seen in 2117. (c) Stereo pair images of the reconstruction calculated at 10 Å resolution, viewed along the twofold symmetry axis. (d) Side view of the sapovirus VP1 dimer.

We observed that our 2117 structure closely resembled a published low-resolution structure of Parkville virus (a sapovirus) [31]. To draw a comparison between vesivirus 2117 capsids and those of the genus *Sapovirus* at higher resolution, we calculated a 3D structure from images of VLPs produced by a chimeric VP1 construct that has been shown to yield high levels of expression [28]. Briefly, a construct consisting of amino acids 1–289 of the Yokote strain VP1 and amino acids 290–560 of the Mc114 sapovirus strain was used to produce VLPs. The chimera consisted of the N-terminal arm, S domain and P1.1 domain of the Yokote strain and the P2 domain, P1.2 domain and VP2 of the Mc114 strain [28]. These particles were imaged in a frozen-hydrated state (Fig. 2a). A total of 2943 particles were picked from 222 micrographs and used to calculate a 3D reconstruction of the chimeric sapovirus VLP at 10 Å resolution (Fig. 2b, c). The sapovirus structure also exhibits typical calicivirus morphology, with notable similarities to that of vesivirus 2117. In particular, the P2 region of the dimeric capsomeres is compact and rounded. The outermost surface of the P2 domain of vesivirus 2117 shows horn-like protrusions at either end, whereas the P2 domains of the sapovirus VLPs appear to have a more curved structure without such large protuberances (Fig. 2d). The S and P1 domains of the two structures appear quite similar at this resolution.

### Structure of FCV at 7 Å resolution

**Fig. 3.**
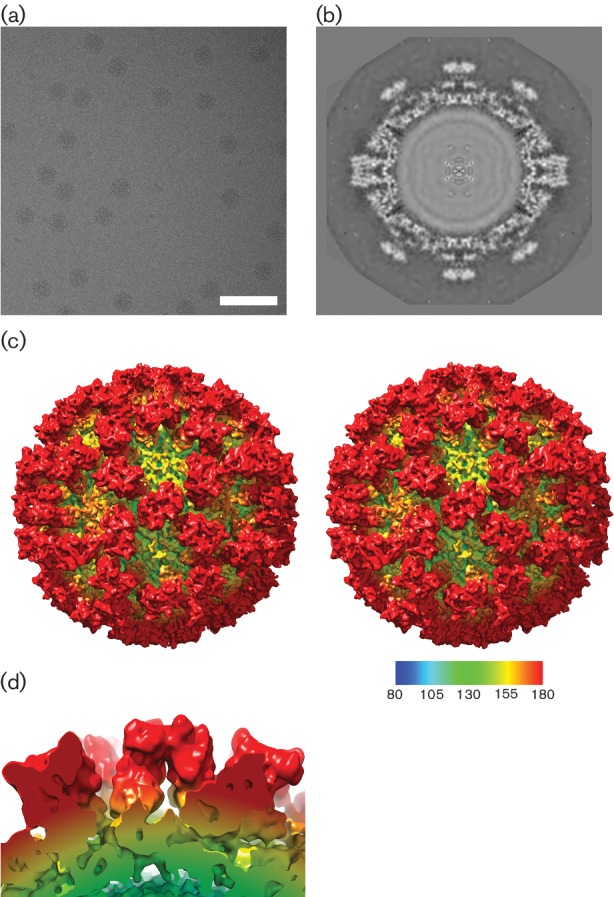
Structure of FCV. (a) Cryo-microscopy of native FCV virions. Bar, 100 nm. (b) The central section shows a broader P domain structure that presents a flatter outer face. (c) Stereo pair images of the reconstruction, calculated at 7 Å resolution, viewed along the twofold symmetry axis. (d) A side view of a dimeric FCV VP1 capsomere.

We have extended the resolution of our structure for the vesivirus FCV strain F9 to 7 Å resolution using a new dataset. A total of 6965 particles were picked from 241 micrographs (Fig. 3a) and used to calculate the 3D reconstruction shown in Fig. 3(b, c). This structure exhibits striking differences from the vesivirus 2117 and sapovirus structures set out above; the FCV P2 region forms a flattened, rhombus-shaped outer face (Fig. 3c, d). Central sections through each reconstruction (Figs 1b, 2b and 3b) show that this region of the P domain of FCV has a broader conformation than that of either 2117 or sapovirus.

### Structural comparison of members of the *Caliciviridae*

**Fig. 4.**
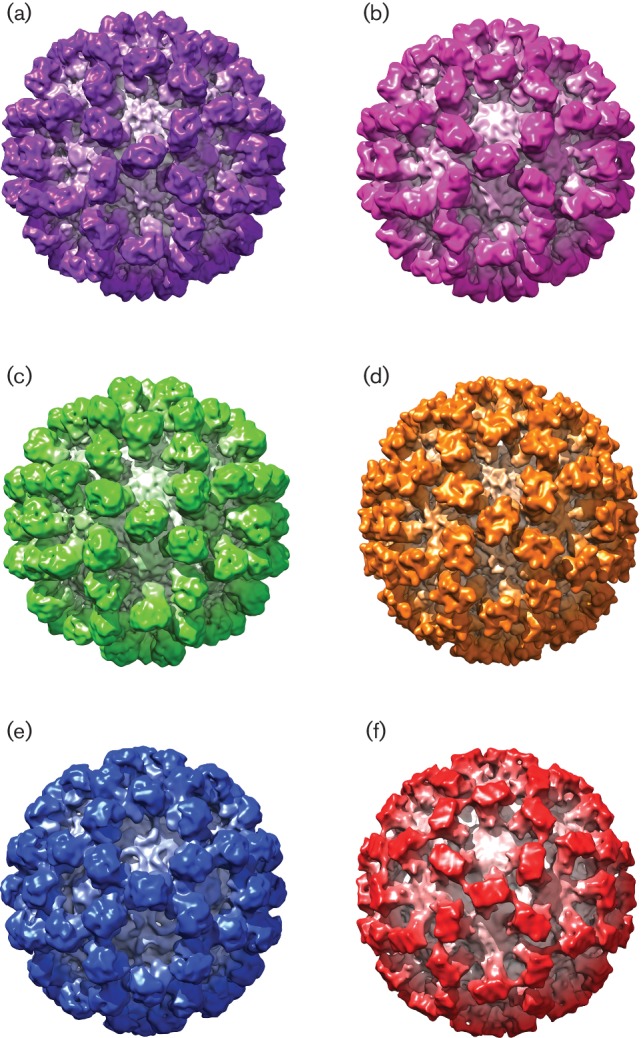
Side-by-side comparison of (a) vesivirus 2117, (b) a chimeric sapovirus, (c) RHDV, (d) FCV, (e) norovirus GII.10 and (f) NoV at 10 Å resolution, viewed along the twofold symmetry axis.

**Fig. 5.**
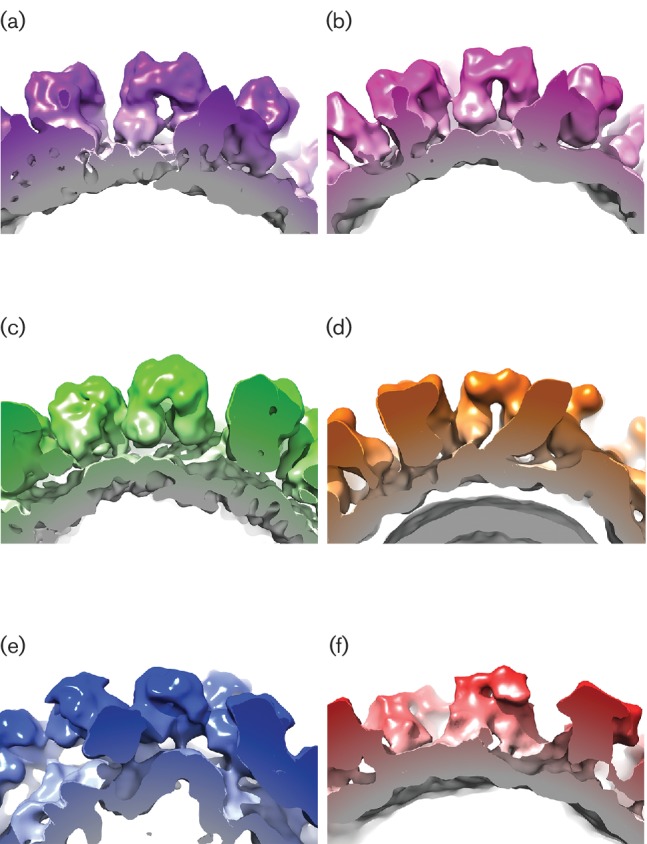
Side-by-side comparison of the P domains of (a) vesivirus 2117, (b) a chimeric sapovirus, (c) RHDV, (d) FCV, (e) norovirus GII.10 and (f) NoV at 10 Å resolution.

Given the clear differences in capsomere morphology between these three structures, we sought to extend our comparison to include a capsid structure from each genus of the *Caliciviridae*. The structures of a lagovirus (RHDV: EMDB 1933; Fig. 4c) and two noroviruses (EMDB 5374 and PDB 1IHM; Fig. 4e, f, respectively) were used to calculate density maps filtered to 10 Å resolution. Likewise, the sapovirus (Fig. 4b) and FCV (Fig. 4d) structures presented above were filtered to 10 Å (the resolution attained for vesivirus 2117; Fig. 4a). Fig. 4 shows a side-by-side comparison of the six 3D structures viewed along the twofold symmetry axis. The similarities (and differences) between the maps are clearly recognized at this resolution. Fig. 5 shows a side-by-side comparison of the P domains of each of the viruses compared in Fig. 4. The P domains of vesivirus 2117, sapovirus and RHDV (Fig. 5a–c, respectively) appear rather similar, showing a compact morphology. The capsomeres of FCV, norovirus GII.10 and NoV (Fig. 5d–f, respectively), however, appear to be quite distinct in conformation; indeed, there are notable differences between the two norovirus structures GII.10 and Norwalk. The P domains of vesivirus 2117, sapovirus and FCV appear to be raised off the S domain, as previously observed for RHDV and MuNV-1, in contrast to the P domains of NoV, which form a more collapsed conformation in relation to the S domain [33, 34].

To provide a quantitative index of similarity between the maps under investigation, the maps were brought to a common alignment, resolution and scale. Cross-correlation coefficients between vesivirus 2117 and each of the other five structures were calculated (Table 1). In such analyses, identical maps would give a correlation of 1.0. The structure that produced the highest correlation value when aligned to the vesivirus 2117 structure was the chimeric sapovirus described here, giving a correlation value of 0.9214. This might be expected given the apparent similarities in the maps when visualized by isosurface rendering as in Fig. 4. It is immediately apparent that the sapovirus structure is the most comparable to that of vesivirus 2117, in both the S domains and the P domains of the VP1 proteins. When fitting the structure of RHDV into that of vesivirus 2117, a correlation value of 0.9007 was recorded, signifying a high degree of similarity between the two capsids, again in both the S and P domains of VP1. These results are unexpected given that all three viruses (vesivirus 2117, sapovirus and RHDV) are classified into different genera of the *Caliciviridae*. When the structure of FCV is fitted into the vesivirus 2117 structure, the correlation value obtained is comparatively low at 0.8478. While this value still indicates reasonable similarity, consistent with a common architecture, we might have expected these two viruses to exhibit a higher degree of correlation, as both FCV and vesivirus 2117 are classified in the same genus, *Vesivirus*. While the NoV structure produced a correlation value of 0.8117, the norovirus GII.10 structure produced a correlation value of 0.8498. This suggests that the structures of FCV and norovirus GII.10 are equally similar to that of vesivirus 2117. When observing the structures in Fig. 4, the P2 domains of norovirus GII.10 seem similar to those of vesivirus 2117, whereas the S domains and P1 domains of FCV seem to resemble those of vesivirus 2117. When comparing the structures side by side, it is likely that different domains of the capsid proteins of the two viruses have contributed to the close correlation values recorded. When the norovirus GII.10 structure is fitted into the FCV structure, a correlation value of only 0.6261 is produced, supporting this hypothesis of different components of the capsids contributing to the similar correlation values.

**Table 1.** Correlation values calculated by docking structures of several calicivirus capsids into that of vesivirus 2117 A correlation value of 1.0 indicates perfect agreement.

Virus structure fitted into vesivirus 2117	Correlation value
A chimeric sapovirus	0.9214
RHDV	0.9007
FCV (F9)	0.8478
NoV	0.8117
Norovirus GII.10	0.8498

### Phylogenetic analysis of vesivirus 2117

**Fig. 6.**
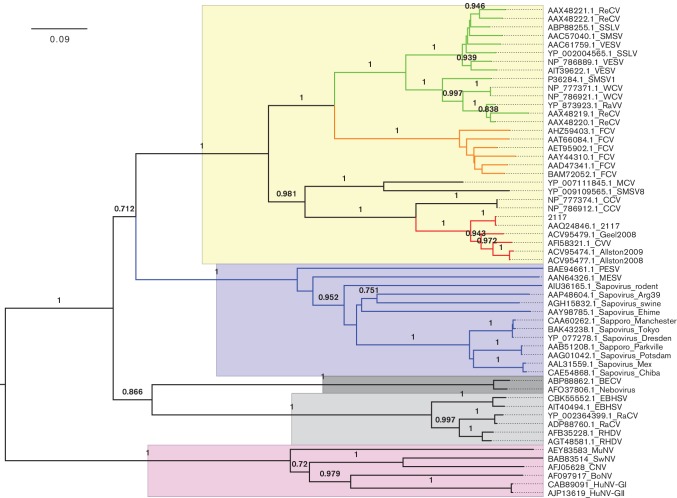
Vesivirus 2117 neighbour-joining tree. The evolutionary history of the VP1 capsid of 2117 was inferred using the neighbour-joining method. The vesivirus 2117 cluster is highlighted in red; FCV cluster, in orange; VESV cluster (includes walrus, reptile and sea lion viruses), in green; and the sapovirus cluster, in blue. The proportions of replicate trees in which the associated taxa clustered together in the bootstrap test (1000 replicates) are shown next to the branches. The tree is drawn to scale, with branch lengths in the same units as those of the evolutionary distances used to infer the phylogenetic tree. The genus *Vesivirus* is highlighted in yellow; the genus *Sapovirus*, in blue; the genus *Nebovirus*, in dark grey; the genus *Lagovirus*, in light grey; and the genus *Norovirus*, in pink. BECV, bovine enteric calicivirus; BoNV, bovine norovirus; CCV, canine calicivirus; CNV, canine norovirus; CVV, canine vesivirus; EBHSV, European brown hare syndrome virus; FCV, feline calicivirus; HuCV, human calicivirus; HuNV-GI, human norovirus GI; HuNV-GII, human norovirus GII; MCV, mink calicivirus; MuNV, murine norovirus; PESV, porcine enteric sapovirus; RaCV, rabbit calicivirus; RaVV, rabbit vesivirus; ReCV, reptile calicivirus; ReVV, reptile vesivirus; RHDV, rabbit hemorrhagic disease virus; SMSV, San Miguel sea lion virus; SMSV1, San Miguel sea lion virus 1; SSLV, Steller sea lion vesivirus; SwNV, swine norovirus; VESV, vesicular exanthema of swine virus; WCV, walrus calicivirus.

Fifty-one sequences of the VP1 capsid protein from the three genera highlighted as being structurally similar to that of vesivirus 2117 (*Vesivirus*, *Lagovirus* and *Sapovirus*) were aligned and analysed to compute a phylogenetic tree (Fig. 6). As expected, this analysis placed vesivirus 2117 firmly within the *Vesivirus* genus and showed that the sapoviruses are more closely related to the vesiviruses than the *Lagovirus* genus. Vesivirus 2117 isolates and canine vesivirus are clearly closely related and occupy their own clade. These viruses along with the canine caliciviruses, SMSV8 and mink calicivirus are distinct from the FCV and VESV clades. Cross-correlation data indicated a high degree of similarity between vesivirus 2117 and both sapovirus and RHDV but a lesser degree when compared with FCV. At 10 Å resolution, gross morphological similarities or differences may not indicate common tertiary structures; therefore, such comparisons should be interpreted with care. However, the P2 domain plays a critical role in the viral infection cycle as it contains both the major immunodominant epitopes and the receptor-binding site [32, 35]. Thus, differences in capsid morphology between the vesivirus clades and similarities between vesivirus 2117 and viruses in different genera within the *Caliciviridae* may allude to important functional differences and similarities, respectively.

## Discussion

Vesivirus 2117 has caused significant financial losses owing to the contamination of bioreactors at Genzyme (Allston Landing and Geel facilities) where CHO cells were used for the production of biopharmaceuticals. The contamination necessitated the shutdown and decontamination of bioreactors, causing a significant delay in the delivery of drugs to patients with Gaucher or Fabry disease (17). The substantial impact of this contamination highlights the need for effective adventitious agent testing.

We have determined the structure of vesivirus 2117 at 10 Å resolution by cryo-EM and 3D reconstruction. We also determined the structure of a chimeric sapovirus at 10 Å resolution and showed the high degree of morphological similarity between the two. Indeed, vesivirus 2117 appeared to be structurally closer to sapovirus and RHDV (a lagovirus) than other vesiviruses, including FCV. However, phylogenetic analysis based on VP1 sequences confirmed that vesivirus 2117 is correctly classified as a vesivirus, also showing that it resides in a clade distinct from FCV and VESV.

The dimeric capsomeres formed of vesivirus 2117 capsid protein present two horn-like structures on the outer surface of the P2 domains. Similar structures are present on the outer faces of P domains of sapovirus VP1, although they are less pronounced. MuNV also exhibits prominent horn-like protrusions on the outer surface of the P2 domain of VP1 [34]. Conversely, the P2 domains of FCV form a rhombus-shaped structure with a flatter outer surface (similar to that of SMSV and Tulane virus [36, 37]). Despite this obvious morphological difference, sequence analysis suggests that FCV is indeed the calicivirus most closely related to vesivirus 2117 for which we have a capsid structure.

The outer face of the FCV capsomere incorporates the receptor-binding site. Two molecules of feline junctional adhesion molecule-A bind to the flat surface in a head-to-tail arrangement [13, 32]. Receptor binding induces conformational changes in the capsid that are hypothesized to prime the virion for genome uncoating in the late endosome. FCV is the only calicivirus that has been shown to enter via attachment to a protein receptor. Sapoviruses and MuNV are thought to bind sialic acid moieties to mediate attachment [38, 39], while RHDV and human noroviruses bind histo-blood group antigens – complex carbohydrates linked to glycoproteins or glycolipids on the surface of erythrocytes and mucosal epithelial cells [40–42]. Our morphological comparison of vesivirus 2117 and sapovirus may therefore provide clues to entry pathways exploited by the 2117 and CCV clades of the genus *Vesivirus*.

There is a paucity of data concerning pathology caused by vesiviruses in the CCV and 2117 clades. Reports of enteritis associated with CCV suggest the possibility of sapovirus-like disease [43], whereas for 2117, the natural host and pathology remain unknown.

Interestingly, the first and second ORFs of vesivirus 2117 are separated by a stop codon, which is typical of the noroviruses and vesiviruses. However, the capsid protein is encoded within the first ORF along with the viral non-structural proteins, typical of the sapoviruses and lagoviruses [16]. This has, to date, only been described for canine calicivirus [44], which is possibly the closest related virus to vesivirus 2117.

While phylogenetic analysis indicated a close genetic relatedness between vesivirus 2117 and canine vesivirus, the striking morphological similarities with sapoviruses led us to question the taxonomic classification of vesivirus 2117 as a vesivirus. However, based on sequence data alone, vesivirus 2117 is undoubtedly more closely related to FCV than it is to the genus *Sapovirus*. Structural analysis has been proposed to provide insights into the common ancestry of distantly related viruses following the discovery of fold conservation between viruses that infect highly divergent branches on the tree of life [30]. Within the *Caliciviridae*, VP1 proteins share a common topology. The fact that the outer face of the P domain presents the receptor-binding site and major immunodominant epitopes means that it is subject to significant evolutionary pressure from immune surveillance. Typically, receptor-binding sites of viruses are embedded in hyper-variable regions of capsid proteins. Thus, morphological differences/similarities in the P domain may be uncoupled from genetic relatedness but may provide important clues concerning critical aspects of virus biology, such as entry pathway.

## Methods

### Virus culture and purification

Vesivirus 2117 VLPs were produced by baculovirus expression of the VP1 gene (lacking the predicted leader sequence) in Hi5 cells. Six days post-infection, cells were freeze–thawed and the lysate was clarified by centrifugation at 14 000 ***g*** for 30 min. The supernatant was filtered (0.45 µm vacuum filter) and protein was precipitated by addition of polyethylene glycol to a final concentration of 10 % followed by incubation at 4 °C. The precipitate was centrifuged at 8000 ***g*** and 4 °C for 30 min. The VLP-containing pellet was then resuspended in boric acid buffer (0.2 M boric acid, 0.5 M NaCl, pH 7.5). VLPs were then centrifuged through a sucrose cushion (30 %, 150 000 ***g*** at 4 °C). The VLP pellets were subsequently resuspended in boric acid buffer, centrifuged (8000 ***g***, 4 °C for 10 min) and mixed with an equal volume of PBS containing 0.5 M NaCl and 4.51 M CsCl for ultracentrifugation using a Sw55 Ti rotor at 40 000 r.p.m. for 20 h at 4 °C. Purified VLPs were then collected and dialysed against PBS.

FCV strain F9 was propagated in Crandell Rees feline kidney cells for 8 h. The virus-infected cells were pelleted from the culture medium by centrifugation (1500 ***g***, 10 min at 4 °C). The pellet was resuspended in TBS (250 mM NaCl and 85 mM Tris/HCl, pH 7.2) and freeze–thawed at −80 °C prior to sonication with an equal volume of Vertrel XF (Sigma-Aldrich). The cell suspension was then clarified by low-speed centrifugation (7000 ***g***, 10 min at 4 °C), and the aqueous phase was subjected to repeated sonication. Virus was then purified from the aqueous phase by centrifugation through a caesium chloride gradient (1.31–1.45 g ml^−1^) using a SW-41 Ti rotor at 28 000 r.p.m. for 8 h at 12 °C. The purified virus was collected from the gradient and dialysed into virion buffer (10 mM Tris, 150 mM NaCl and 20 mM MgCl_2_, pH 7.2).

Sapovirus particles were made using a baculovirus expression system in Tn5 cells as previously described [27].

### Electron microscopy

Vesivirus 2117 VLPs and FCV virions (4 µl) were loaded on-to glow discharged C-flat holey carbon support films (R2/2 ProtoChips), blotted at 4 °C for 4 s at 100 % humidity and plunged into liquid-nitrogen-cooled liquid ethane using an FEI Mark IV Vitrobot. Vitrified samples were imaged in a JEOL 2200 FS cryo-microscope equipped with a Gatan 626 cryo-stage. Energy-filtered images were recorded (with a 20 eV slit width) on a Gatan Ultrascan US4000 charge-coupled device camera at a magnification of ×100 000, corresponding to a pixel size of 1.05 Å per pixel (vesivirus 2117) or a Direct Electron DE20 direct detection device at a magnification of ×40 000 with a pixel size of 1.39 Å per pixel (FCV). Sapovirus VLPs were plunged using the same method but using R1.2/1.3 Mo 200 mesh holey carbon grids (Quantifoil), imaging was performed in a JEOL 2200FS at a magnification of ×80 000 and micrographs were recorded on a TVIPS 4k×4k CCD camera, giving a pixel size of 1.6 Å per pixel.

### 3D image reconstruction

Two hundred and forty-two micrographs of vesivirus 2117 VLPs, 241 micrographs of FCV and 222 micrographs of SV VLPs were processed to calculate 3D reconstructions. Images of particles were contrast transfer function corrected and extracted from micrographs using the BSoft program Bshow [45]. The vesivirus 2117 and SV particles were masked and sorted by size into five classes by cross-correlation against fuzzy ring models using spider [46]. The most populous class for each was selected for use in further processing. The origins and orientations of the particles were then determined using the polar Fourier transform method (PFT2), and the EM3DR2 program was used to create 3D reconstructions [47, 48]. Resolution estimates for each map were determined by dividing the data set into two and calculating 3D reconstructions from each half. The paired reconstructions were compared in Bresolve to compute a number of indices of similarity including the Fourier shell correlation (FSC). A FSC cut-off value of 0.5 was taken (Fig. S1, available in the online Supplementary Material). To draw comparisons between different calicivirus capsids, additional structures were downloaded from the Protein Data Bank and Electron Microscopy Data Bank public databases. Density maps were calculated from Protein Data Bank files using the eman program pdb2mrc. All density maps were then low pass filtered to a common resolution of 10 Å using eman [49]. UCSF Chimera was used for visualizing cryo-EM reconstructions (using an isosurface threshold of the mean plus 1 sd) and for calculation of correlation values between pairs of maps. Correlation values were calculated in UCSF Chimera using the ‘Fit in Map’ function [50].

### Phylogenetic analysis

Our 2117 capsid amino acid sequence was used as a query sequence in a blastp search against the GenBank non-redundant database. The top hit was to an existing 2117 sequence (AAQ24846; 99 % identity), followed by three hits to other suspected 2117 sequences sampled in Geel, Belgium (ACV95479; 85 % identity) and Allston, USA (ACV95474, ACV95477; 84 % identity). The next most similar hits were canine vesivirus (AFI58321; 84 % identity) and canine calicivirus (NP_786912, NP_777374; 68 % identity) sequences. All of the above sequences had an *E* value of 0 and were selected for inclusion in the phylogenetic analysis. Representative sequences from other species within the genera *Vesivirus*, *Sapovirus*, *Lagovirus* and *Norovirus* of the family *Caliciviridae* were also selected from the blast hits for inclusion in the phylogenetic analysis. In total, 57 protein sequences were selected (including our 2117 capsid sequence) and aligned using clustalw within the mega software [51]. The evolutionary history of the 2117 capsid sequence was inferred using the neighbour-joining method (1000 bootstrap replicates) to generate a phylogenetic tree, evolutionary distances were computed using the JTT-matrix-based method and all positions in the alignment containing gaps and missing data were eliminated, leaving a total of 402 positions in the final dataset.

## Supplementary Data

148 Supplementary File 1
