# Supplementary material for: Vesivirus 2117 capsids more closely resemble sapovirus and lagovirus particles than other known vesivirus structures
Source: J Gen Virol. 2017 Mar 17;98(1):68–76. doi: 10.1099/jgv.0.000658 (PMC5370393; doi:10.1099/jgv.0.000658)

Supplementary Figures

Figure S1: Fourier Shell Correlation plots for the three dimensional reconstructions of vesivirus 2117 (A), a chimeric sapovirus (B) and feline calicivirus (C).

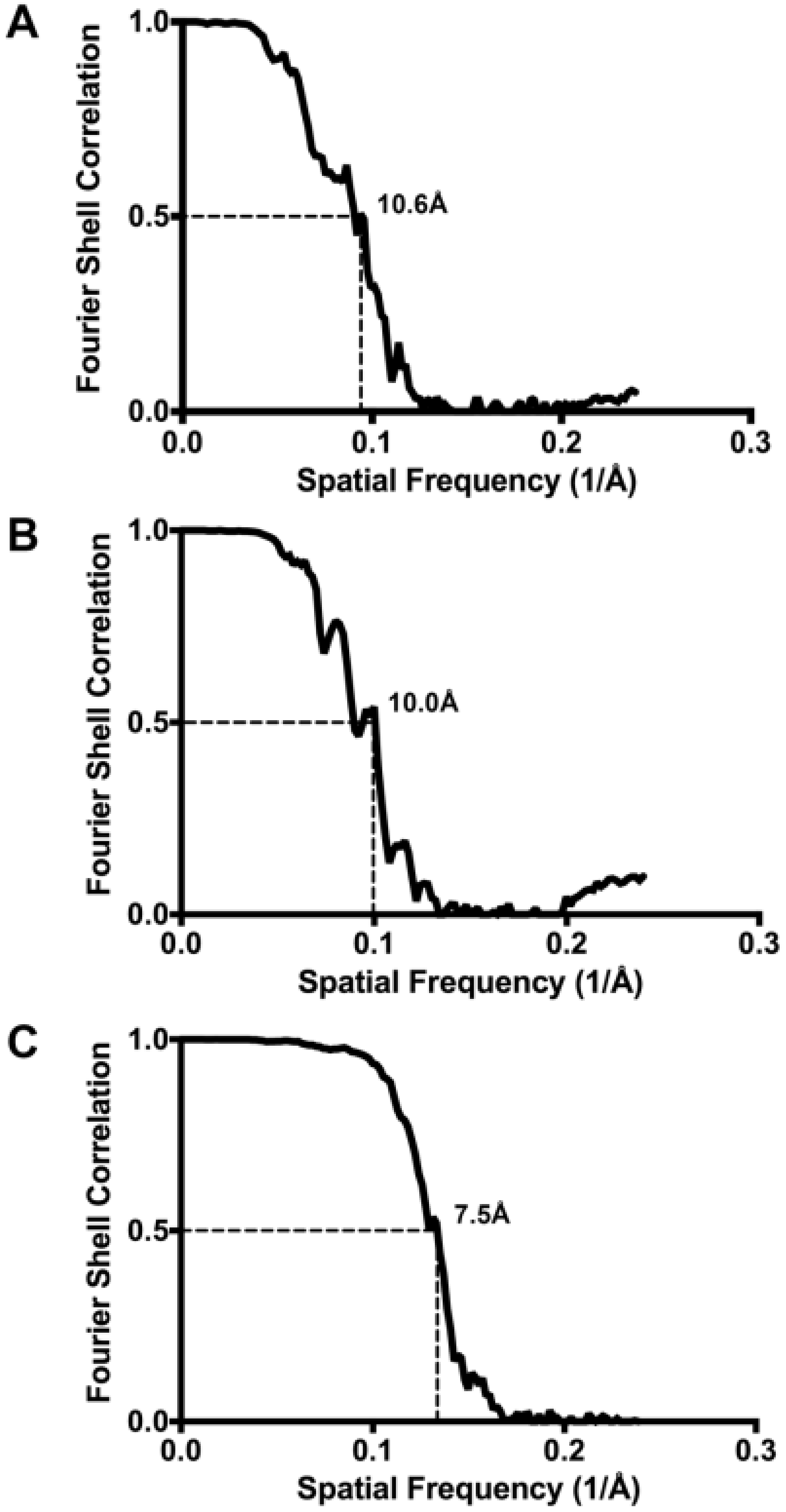

Supplement: Supplementary File 1 [file jgv-98-68-s001.pdf]
